# Management Strategies for Lamb Production on Pasture-Based Systems in Subtropical Regions: A Review

**DOI:** 10.3389/fvets.2020.00543

**Published:** 2020-09-15

**Authors:** Cesar Henrique Espírito Candal Poli, Alda Lucia Gomes Monteiro, Thais Devincenzi, Fernando Henrique Melo Andrade Rodrigues de Albuquerque, Juliano Henriques da Motta, Luiza Ilha Borges, James Pierre Muir

**Affiliations:** ^1^Departamento de Zootecnia, Faculdade de Agronomia, Universidade Federal do Rio Grande do Sul, Porto Alegre, Brazil; ^2^Departamento de Zootecnia, Universidade Federal do Paraná, Curitiba, Brazil; ^3^Programa Nacional de Producción de Carne y Lana, Instituto Nacional de Investigación Agropecuaria (INIA), Tacuarembó, Uruguay; ^4^Embrapa Caprinos e Ovinos, Sobral, Brazil; ^5^Texas A&M AgriLife, Stephenville, TX, United States

**Keywords:** sheep, concentrate supplement, pasture management, performance, ewe

## Abstract

Sheep production on pasture plays an important role in subtropical climates around the world, with great economic and environmental relevance to those regions. However, this production is much lower than its true potential in subtropical regions, largely due to lack of knowledge of how to feed grazing lambs, and mitigate gastrointestinal parasite infections. Due to weather instability and the high growth rate of tropical grasses, it is difficult to adjust the quality and quantity of feed consumed by lambs. In addition, due to warm, wet weather during spring, summer, and autumn, gastrointestinal parasite infection can be intense on subtropical pastures. Thus, the objective of this paper is to summarize 17 years of research in southern regions of Brazil testing alternative management for sheep farmers under these challenging conditions. Our review indicates that ewes play important roles raising their lambs. Besides protecting and providing milk, they leave a better pasture structure for lamb nutrition. The use of creep feeding and creep grazing are additional alternatives to improve lamb growth. However, feeding supplementation with concentrate can deteriorate pasture quality at the end of the summer–autumn season. Gastrointestinal parasitic infections can be reduced with improved lamb nutrition, although L3 larvae of *Haemonchus contortus* can be present at various pasture heights. This indicates that it is difficult to control L3 ingestion solely by manipulating grazing heights. We summarize important technologies for raising lambs on pasture-based systems to make the best of high herbage growth and minimize intense parasitic infections common in subtropical regions. We discuss research results in light of the latest studies from other ecoregions and climates, although there is a lack of similar research in subtropical regions of the world.

## Introduction

Sheep production plays an important role in subtropical climates around the world as exemplified by Australia, Brazil, China, South Africa, Spain, and Uruguay. This occurs both at the subsistence and commercial levels for meat and wool. Today, lamb meat production is growing in importance, emphasizing the need to understand its potential economic and environmental impact within production systems around the world. Subtropical pastures are often characterized by two distinct sets of forage species: one that thrives in the warm season and another completely different group that grows in a cool season characterized by frosts and short-duration freezes. This requires different technology for each season whether the warm- and cool-season forage species share the same pasture at different times or grow in distinct paddocks.

To optimize lamb production sustainably, it is necessary to understand key animal/environment dynamics. In subtropical areas, it is possible to raise lambs with either temperate, tropical pastures or both pasture types. This pasture growth is associated to relatively high rainfall in southern Brazil [1,200–2,100 mm; (1)] distributed along the year. This gives subtropical pastures great potential for low-cost lamb production based on pasture. However, due to weather instability, it is difficult to adjust lamb diet quality and quantity (2) and to control gastrointestinal parasite infection (3, 4). Because of high humidity and temperature, highly productive tropical pasture can meet lamb nutritional needs and generate complex canopy structures that need to be properly understood for sheep production. The challenge is to make the best of pasture growth for raising lambs in this environment.

There are key technical issues related to grazing sheep supplementation, sheep gastrointestinal parasite control, and the economic aspects of sheep production in subtropical regions. These have been studied in the southern region of Brazil. Our objective was to summarize 17 years of research carried out by the Sheep and Goat Production and Research Center (LAPOC) at the Universidade Federal do Paraná and by the Sheep Production Teaching and Research Center (CEPOV) at the Universidade Federal do Rio Grande do Sul. Our results will be compared with those of the most recent studies reported in other ecosystems and climates.

## Grazing Sheep Management and Supplementation

### Weaning and Concentrate Supplementation

Subtropical areas can support both temperate and tropical pastures in overlapping seasons that provide forage year-round, begging the question “why do producers feed concentrates to sheep?” One possible answer is that, despite the lower costs of pasture-based systems, these can carry high risks. These are associated to the fact that flock managers have limited control of feed production and gastrointestinal nematode (GIN) infestations (3). Efforts to produce younger slaughter lambs (3–6 months old with a minimum live weight of 30 kg) when there is a price incentive and energy deficits during periods of greater flock nutrient requirement also contribute to additional pasture-management challenges.

Feed supplementation can affect grazing sheep in different ways, and the understanding of all the complex variables involved is still not completely known. Detailed reviews about the effects of supplementation on grazing sheep were compiled by Dove (5), Clark and Woodward (6), and Kenyon and Webby (7). However, some main effects of supplementation on grazing sheep in subtropical region can be listed: (1) type of supplement (8, 9); (2) level of supplement (10, 11); (3) type of pasture (8); (4) type of animal (12); and (5) animal feeding system (13). One of the most important feed supplement effects is related to the energy/protein ratio of the animal diet. When energy or protein fails to meet animal requirements, ruminants will only respond to the one that is limiting (14). In addition, one supplement compound can affect the digestibility of other compounds (15). Highly degradable carbohydrate can, for example, decrease fiber digestibility. The amount of concentrate supplied to the animal can also modify lamb performance. Aguerre et al. (10), for example, found that supplementing grazing lambs with greater amounts of sorghum grain resulted in higher ruminal fermentation rates that reduced fiber digestibility and total organic matter intake.

In addition to the supplement quantity and quality, the way it is provided to the animal has an important influence on its performance. Supplementing sheep with grain on pasture can promote a more variable rumen pH than if it is given as a total mixed diet, thereby affecting lamb digestion and growth (10). High feeding rates can also increase the passage rate through the rumen, decreasing overall feed digestibility (15). All these effects can also be influenced by animal genetic characteristics. Amino acid requirement of an animal, for example, is related to its performance level (12). Greater genetic lamb performance potentials require more amino acids than that provided by the milk from the mothers.

In the majority of cases, when concentrate supplement is fed to sheep on pasture, less forage is ingested. The animal will often replace forage with concentrate, but the rate of substitution depends on the amount and type of concentrate ingested (11). According to Garcés-Yépez et al. (16), the more the sheep are supplemented with starch, the higher the forage substitution rate becomes. Supplements with less starch and more digestible fiber promote a less substitution rate. However, this rate varies according to the level of supplement provided to the animals. Garcés-Yépez et al. (16) did not find effect on Bermudagrass hay intake when lambs were supplemented with concentrate at a low feeding level (0.4–0.5% of LW); only when they were supplemented at a higher level (0.8–1% of LW) did forage intake decreased. Ideally, supplement should increase forage intake by maximizing forage digestibility and passage rate; otherwise, it becomes a substitute rather than a supplement. To minimize substitution and maximize forage use efficiency, the first step is to identify what the pasture has to offer and what limits forage intake. Supplement content and quantity can then be tailored to the flock's need such that it serves as a complement to pasture rather than a substitute (17).

Concentrate formulation designed to supplement pasture-based flocks in subtropical regions should therefore seek to supply minerals, energy, and protein to overcome deficiencies in the forage (18). Otherwise, animal performance and pasture utilization may not meet production goals. One of our studies (13), for example, offered concentrate at 1% of the animal liveweight (LW) to lambs nursing on ewes grazing good-quality tropical pasture (10% crude protein and 55% total digestible nutrients) but did not achieve the expected benefits (Table 1). We did not find a performance difference between supplemented and control animals, concluding that the nutrients provided by the pasture and the lactating ewes were sufficient to keep lambs at performance levels equivalent to lambs supplemented at 1% LW. Subsequent studies (11, 20, 21) indicated that, in that type of pasture, lamb supplement must be above 2% LW to induce measurable differences vis-á-vis control animals.

**Table 1:** Unweaned lamb slaughter weight and age as well as average daily gain (ADG) of finishing systems on summer pasture from subtropical region of Brazil.

**Concentrate supplementation**	**Pasture**	**Season**	**Forage (kg/ha)**	**Canopy (cm)**	**Leaves (kg/ha)**	**Leaf:stem**	**Lamb (kg/ha)**	**Slaughter weight (kg)**	**Age at slaughter (d)**	**ADG (g)**	**References**
**Unweaned and pasture finished**											
Exclusively pasture	Tifton-85	Oct-Jan	5,828	27.0	2,130	0.66	14.2	32.0	101	281	(13)
Exclusively pasture	Tifton-85	Nov-Mar	3,247	13.3	1,043	0.69	33.0	32.7	136	135	(19, 20)
**Unweaned, finished in pasture, creep supplemented**											
1% LW DM/d	Tifton-85	Oct-Jan	3,709	23.9	1,275	0.60	9.75	32.0	105	282	(13)
2% LW DM/d	Tifton-85	Nov-Mar	3,554	14.0	1,049	0.65	34.0	37.3	136	275	(19, 20)

*DM, dry matter; LW, live weight*.

Our studies also focused on lamb supplement effect on pasture canopy structure. Fajardo et al. (11) reported that the level of concentrates fed to lambs to meet NRC (22) recommendations had a deleterious effect on pasture canopy structure when fed during summer–autumn. Offering this supplement during lamb finishing favored forage inflorescence and taller plants. They recommended that supplements fed to lambs on upright tropical grass pastures should be avoided during grass inflorescence if seed production is not a priority. Large bulk grazers mixed with or following more selective sheep might also keep upright grass in vegetative growth since less selective species such as bovids or equids are more likely to ingest fibrous inflorescences (23).

A recent publication described the effect of a lamb feed system on Bermudagrass (*Cynodon* spp.) cv. Tifton-85 sward canopy structure. Silva et al. (24) compared four treatments: unweaned lambs with no supplement, unweaned lambs supplemented with concentrate using creep feeding, weaned lambs not supplemented, and weaned lambs supplemented with concentrate. Weaning caused a greater presence of leaves and stems in all sward strata, which increased when concentrate supplementation was fed to the lambs. Independent of supplementation to the lambs, the systems without weaning fostered a sward structure with a greater leaf/stem ratio, showing that the ewes have an important role of leaving a better sward structure to the lambs. This study also showed, similarly to what was found by Fajardo et al. (11) with an upright tropical grass, that the supplementation of weaned lambs on Tifton-85 had a deleterious effect on sward structure. Concentrate supplementation can reduce the leaf/stem ratio due to the reduction of grazing time and the increase in diet selection for leaves. However, these results observed in the subtropical region are not always found in other ecosystems. Bosing et al. (25), for example, studying grazing sheep performance on the semiarid grassland steppe of northeastern Asia, reported that supplement (250 g/day) prolongs pasture use duration. Contrary to what we observed in subtropical areas, sheep supplementation in semiarid areas allows reduction of stocking rate due to improved animal performance, allowing greater animal LW gain and pasture growth.

Besides favoring performance, interactions between supplement and pasture can increase sheep productivity per area. For example, reduced forage consumption per lamb as a result of supplements can increase pasture carrying capacity and productivity (11). This can be more important to small and medium producers that have limited access to land such that intensifying use of what land they do have may result in economy of scale. However, benefits are accrued more for weaned lambs or aging ewes. For example, in our studies in southern Brazil [(13, 26, 27), Tables 1, 2], creep feeding lambs at 1% LW/day did not increase pasture carrying capacity because their daily forage consumption was negligible vis-á-vis the lactating ewes.

**Table 2:** Unweaned lamb slaughter weight and age as well as average daily gain (ADG) of finishing systems on Italian ryegrass pasture from subtropical region of Brazil.

**Concentrate supplementation**	**Season**	**Forage (kg/ha)**	**Canopy (cm)**	**Leaves (kg/ha)**	**Leaf:stem**	**Lamb (kg/ha)**	**Slaughter weight (kg)**	**Age at slaughter (d)**	**ADG (g)**	**References**
**Unweaned and pasture finished**										
Exclusively pasture	Aug-Jan	2,815	17.5	807	1.16	8.4	31.7	105	303	(26, 27)
Exclusively pasture	Sept-Dec	4,395	16.6	979	0.69	9.0	34.0	106	204	(28)
**Unweaned, finished in pasture, creep supplemented**										
1% LW DM/d	Aug-Jan	2,971	16.3	725	1.24	8.8	32.4	106	294	(26, 27)
2% LW DM/d	Sept-Dec	3,863	14.7	795	0.71	8.0	33.0	90	307	(28)
**Unweaned, finished in pasture, creep supplemented**										
White clover	Sept-Dec	3,923	15.9	838	0.75		33.2	94	274	(28)

*DM, dry matter; LW, live weight*.

Very few studies have investigated the effect of supplementation and lamb weaning on grassland structure in other subtropical or tropical regions. However, Pullin et al. (29) found that weaning alters the lamb feeding behavior. Lambs that remain with their dams spend less time grazing. In addition, Evan et al. (30), working in a different region and forage species, also agreed with our results that it is difficult to maintain sward height after weaning, particularly toward the end of the grazing period. These studies reinforce our conclusion that early weaning of lambs in subtropical pasture should be avoided. In this scenario, creep feeding, and creep grazing turn out to be important management tools for grazing lambs.

#### Creep Feeding

The response of lamb nutrition to creep feeding can be affected by different factors such as ewe milk production, level and composition of the supplement, and animal genetic characteristics. Wilson et al. (31) showed that creep feeding did not affect milk production. However, creep feeding allows the lamb greater energy and protein ingestion and better performance (31, 32). In fact, the effect of creep feeding on ewe milk production should be better studied in a more challenging environment where the ewe nutritional level is deficient. Creep feeding is an important management strategy to improve lamb performance when it provides greater lamb nutrient ingestion (19). However, as mentioned before, this greater response needs to complement pasture quality (17); otherwise, the supplement becomes redundant (13). Monteiro et al. (21) observed greater lamb performance when grazing Italian ryegrass mixed with Tifton-85 if the level of a balanced concentrate provided in creep feeding was above 2% LW. This response also depends on animal genetic characteristics. Lambs selected for greater growth respond more to supplementation in creep feeding (33, 34). Lamb genetics is even more important when different kinds of amino acid are provided in creep feeding. There is a market response only when lambs are genetically more dependent on high diet quality (12). A similar study carried out in South Africa (35) showed that balancing limiting aminoacids through the concentration supplement using creep feed can potentially increase lamb performance. However, the author observed that this is economically viable only in more intense production systems due to high feeding costs.

Greater carrying capacity can apparently compensate, to an extent, for lower average daily gain (ADG) of lambs weaned and finished exclusively on pasture compared to unweaned animals [(13, 26, 27), Tables 1–4]. Despite differences in stocking rates partially compensating for animal production decline, weaning may not offer efficient economic compensation. Our studies showed that weaning lambs at around 60 days on pastures can increase losses due to GIN infestation, reaching 20% higher mortality compared to unweaned lambs. Despite greater stocking rates, early weaning on pasture may limit final productivity.

Reduced unsupplemented weaned-lamb performance arises from a metabolic profile that reflects inadequate nutrition, namely, low blood glucose and albumin (36). Once this becomes chronic, animals consume insufficient energy and protein to meet their nutritional needs to reach slaughter weights in a timely manner. This indicates that early weaning for finishing lambs on pasture is not a viable tool. In addition, as mentioned before, we observed that ewes leave a better pasture for the lamb to graze (13), with more leaves and fewer stems than when the lambs were weaned by physical separation. Supplementing lambs post early weaning with a concentrate may rectify this situation. Our research confirms that supplementing lambs is likely needed to compensate for nutritional deficiencies and stress resulting from early weaning (Tables 3, 4).

**Table 3:** Weaned lamb (40 or 60 days (d) old) slaughter weight and age as well as average daily gain (ADG) of finishing systems [providing or not balanced [according to NRC (22)] concentrate supplementation of 2% of live weight (LW)] on warm-season pasture from subtropical region of Brazil.

**Supplement/weaned age**	**Pasture**	**Season**	**Forage (kg/ha)**	**Canopy (cm)**	**Leaves (kg/ha)**	**Leaf:stem**	**Lamb (kg/ha)**	**Slaughter weight (kg)**	**Age at slaughter (d)**	**ADG (g)**	**References**
Exclusively pasture 60 d	Tifton-85	Oct-Jan	5,670	24.3	1,950	0.66	18.3	32.0	131	107	(13)
Exclusively pasture 40 d	Tifton-85	Nov-Mar	4,170	18.1	1,235	0.61	100.0	21.1	136	57	(20)
2% LW 40 d	Tifton-85	Nov-Mar	4,774	20.0	1,340	0.56	134.0	34.3	136	152	(20)

*DM, dry matter; LW, live weight*.

**Table 4:** Weaned lamb (40, 42, or 60 days (d) old) slaughter weight and age as well as average daily gain (ADG) of finishing systems (providing or not balanced [according to NRC (22)] concentrate supplementation of 1%, 2% of live weight (LW) or *ad libitum*) on cool-season Italian ryegrass with and without supplements from subtropical region of Brazil.

**Supplement/weaned age**	**Season**	**Forage (kg/ha)**	**Canopy (cm)**	**Leaves (kg/ha)**	**Leaf:stem**	**Lamb (kg/ha)**	**Slaughter weight (kg)**	**Age at slaughter (d)**	**ADG (g)**	**References**
Exclusively pasture 40 d	Aug-Jan	2,900	20.9	1,182	1.08	31.5	31.5	160	115	(26, 27)
Exclusively pasture 42 d	Aug-Jan	3,226	19.3	1,101	0.54	45.3	32.0	198	69	(26, 27)
1% LW 42 d	Aug-Jan	3,794	19.4	1,241	0.50	29.7	32.0	153	106	(26, 27)
2% LW 42 d	Aug-Jan	3,584	19.6	1,153	0.49	36.5	32.0	137	151	(26, 27)
*ad libitum* 42 d	Aug-Jan	3,589	22.5	1,198	0.51	54.5	32.0	107	263	(26, 27)

*DM, dry matter; LW, live weight*.

On cool-season annual ryegrass pastures, increasing concentrate supplement from 1% to *ad libitum* (an estimated 3.2% LW/d) increases lamb ADG and therefore decreases slaughter age vis-á-vis unsupplemented animals (21, 37). Those fed *ad libitum* gain 263 g/day, which meant that they reached their 32-kg slaughter weights at 107 days after birth (DAB), 41 days after weaning (Table 4). Depending on economic returns, concentrate supplement could be recommended for finishing lambs if they are maintained on those pastures. When lambs are supplemented using creep feeding, the negative effect of the supplement on sward structure is minimized by the presence of ewes that can regulate pasture regrowth.

Numerous studies (38–44) tested the effect of lamb supplementation by creep feeding on animal performance and GIN infection. They all showed that creep feeding can be used to increase the lamb LW rate of gain and reduce GIN parasitic infection. The supplementation of suckling lambs with creep feed can also improve lamb dry feed intake and rumen development, leading to earlier weaning (45). The earlier a lamb has contact with solid feed, the sooner it will be a fully functional ruminant (46). However, as mentioned before, it is important to be careful that the amount of concentrate provided to the animal complements rather than substitutes nutrients that the pasture provides; otherwise, it would not likely be economically sustainable.

#### Creep Grazing

Enabling nursing lambs to graze pastures ahead of ewes or allowing them exclusive access to forage banks is called creep grazing. The idea is to allow lactating lambs exclusive access to high-quality forage such as young regrowth, legumes, or other highly digestible, protein-rich plants that ewes cannot reach. In a trial using the legumes *Medicago sativa* and *Lotus corniculatus*, creep-grazing lambs gained 223 g/day, 38.5% more than unsupplemented control animals and the same as those fed with a supplement (47). In these situations, supplement ideally does not substitute for dam milk but, rather, complements it (48).

In New Zealand, creep grazing with protein-rich forage species such as ryegrass/white clover is used to suppress GIN in lambs (49). Sykes and Coop (50) explained that protein supplied through pasture affects sheep ability to respond to infection and may be a useful tool to minimize dependence on chemical methods of parasite control. Such targeted creep grazing might be useful in suppressing GIN in warmer subtropical climates, such as southern Brazil, during lamb finishing. In research at LAPOC, Salgado et al. (51) evaluated GIN infection and body condition score (BCS) of lambs in different production systems. Unweaned lambs allowed to creep graze (free access to *Trifolium repens*) and creep feeding (2% of LW/day of a balanced concentrate) had the best performance. Lambs weaned at 60 days on pasture with no supplement had the lowest performance (live weight gain, FAMACHA, and BCS). Endoparasite infection control and the nutritional status of the lambs were positively influenced by the production system, mainly when they were not weaned and/or received concentrate supplementation on pasture.

The importance of creep grazing for lamb production worldwide has been recognized for more than 60 years (52). Creep grazing has been mentioned not only as an additional feed (53) but also as a management tool for controlling parasitic infection in lambs (54). However, very few studies have been carried out in subtropical regions with grazing ewes using tropical grass species. Our research indicated that this technique can be very useful not only to improve lamb ADG and parasitic control but also to provide high-quality forage in periods with low pasture availability such as between winter and spring periods in the subtropics [(28), Table 2]. That period typically has cool-season forages in decline with slow initial warm-season pasture growth. This period is also associated with the need for good-quality forage because of potential lamb development, a reflection of the autumn breeding period (28).

Our research (28) demonstrated a potential for using creep grazing to finish lambs during periods in which forage quantity and quality do not meet animal requirements. In creep grazing areas, clover herbage mass reached 2,500 kg DM/ha with 20% crude protein and 75% total digestible nutrients, 25 and 10% greater, respectively, than the Bermudagrass pasture in which ewes grazed. The ADG of creep grazing lambs was similar to those lambs fed with 2% LW in concentrate formulated according to NRC (22) and superior to that obtained by unsupplemented lambs (Table 2). Creep grazing also reduced the negative seasonal effects of spring forage slump. Despite the demonstrated potential of clovers for creep grazing lambs, this management tool is not utilized in many tropical and subtropical regions around the world (55).

Stivari et al. (56), at LAPOC, observed a similar initial economic response between creep grazing and concentrate supplementation. Although there is an initial cost of setting up additional fencing for creep grazing, lambs will eventually pay more economic dividends over time. This return may vary with each pasture system and the cost of concentrate vis-á-vis fencing, fertilizer, and seed.

### Gastrointestinal Parasites

In subtropical regions, sheep often face health challenges while on pasture, including gastrointestinal nematodes (GINs), such as *Haemonchus contortus*, ingested as larvae on forages (57). Humid, warm-climate pastures offer favorable growth conditions for several GIN species that develop and survive to the infective L3 stage (58). Controlling pasture height to limit GIN L3 ingestion by sheep might not control the infection under these subtropical conditions as well as it does under temperate pastures and mixed cattle-sheep grazing (59, 60). In a study developed at CEPOV, Tontini et al. (3) found infective larvae L3 of *Haemonchus* spp. at different heights of tropical upright grass, from soil to the leaf tip, during summer–autumn. Similar results utilizing tropical grass species were also found by different authors (61–64) proving that sward height control has limited action against parasite infection in tropical pastures. Adjusting pasture height may be more important for temperate conditions (65, 66). Santos et al. (64) explained that high rainfall and air temperature favors the migration of L3 from feces to grass blades. In contrast to tropical pasture grown in a subtropical region, Pegoraro et al. (67) assessed the number of L3 GINs in a cool-season pasture of Italian ryegrass at CEPOV in southern Brazil and found that most of the L3 were below 5-cm height, and low sward height resulted in greater L3 GIN intake by sheep.

Rotating sheep through pasture may interrupt GIN reproductive cycles; however, rest intervals needed between grazing periods may be too long to be practical. This can reduce parasitism in a temperate climate but comes with an overall negative effect on animal performance, including less ADG (68) and sometimes no economic advantage (69). Some GIN larvae hatch and develop into infective L3 within days of deposition and can survive for up to 9 weeks in warm conditions but longer in cooler weather (70) and climates (71). Almeida et al. (72), working with *H. contortus* in humid subtropical pastures in southern Brazil, found that 322, 350, 294, and 182 days were required for *Urochloa decumbens* pasture to be L3-free in autumn, winter, spring, and summer, respectively. Such long rest periods, especially in subtropical and tropical regions, will likely result in poor herbage quality due to rapid plant maturation once sheep are rotated back onto pasture. Shorter rests, normally 21 to 28 days, result in better animal nutrition, but most warm-climate studies indicate that rests of 31+ days are required to even begin lowering GIN L3 viability (73, 74) and 182 to 350 days, depending on season, to completely free the pasture of infection (72). Research by Smith et al. (75) indicated that, if rotations are short, continuous grazing may result in lower GIN infection because grazers are allowed to selectively feed farther from feces in pasture.

As already mentioned, several studies have shown that creep feeding and creep grazing can be an important tool to reduce lamb GIN infestation and can be very useful for subtropical regions (51). In addition to high nutrition demand of lambs and the favorable environment for pasture contamination, lactating ewes are also an important source of infective larvae (76, 77). In fact, these tools can potentially help lambs overcome this challenge through the improvement of animal nutrition and immunity (78). Therefore, creep feeding and creep grazing can be one of the best management strategies for reducing the lamb GIN in subtropical regions.

### Economic Aspects of Sheep Production Based on Pasture in Subtropical Regions

Economic evaluation of Brazilian lamb finishing systems carried out at LAPOC (79, 80) confirms that lamb finishing based on pastures generally provides greater returns than when feed concentrate is used, especially when lambs are not weaned early. Stivari et al. (56) found that nutrition is the production factor that most influenced lamb-finishing costs on pasture, independent of supplement strategy. In another study at LAPOC, Stivari et al. (81) compared six scenarios to evaluate the economic feasibility of creep grazing or creep feeding finish lambs. They compared forage allowance of 12 or 8% LW DM/day and the percentage of *T. repens* supplement pasture area (30–50% relative to the primary pasture area). The creep grazing finishing system with 8 or 30% of *T. repens* as well as the creep feeding system (concentrate fed at 2% of LW/day/ha) with 8% LW of forage allowance promoted the best short-term economic results.

Research efforts in southern Brazil's subtropical regions have focused on developing techniques to finish lambs on Tifton-85 and *Panicum maximum* cultivar IZ-5 (common name: Aruana grass) summer pasture (Tables 1, 3). Efforts in winter pastures have focused on Italian ryegrass [(26–28, 79, 80), Tables 2, 4]. The results of these studies summarize the importance of pasture system to lambs, whether they are weaned or not. When lambs were still nursing, ADG reached 190 g at 124 DAB on tropical grasses and 226 g at 115 DAB during winter with Italian ryegrass. These were superior (*P* ≤ 0.05) to weaned lambs that gained 87 g ADG on the same pasture. Nursing lambs reached target slaughter weight (32 kg LW) at 117 DAB, similar to lambs fed in confinement (21). Considering the favorable results from unweaned lambs, this simple technique has the potential to lower costs while maintaining productivity and animal well-being resulting from lower lamb stress (19). However, in a system without weaning and 8 months between lambing and slaughtering, ewe recovery time may be too short prior to the next breeding period. In this situation, forage quantity and quality become even more important.

## Conclusions

Besides providing milk and reducing weaning stress, grazing ewes can leave a higher-quality pasture canopy structure for lambs. The amount and type of concentrate supplemented to the lambs can also compensate for lower herbage quality, regardless of canopy structure, and can be adjusted according to pasture characteristics. In turn, concentrate supplementation can indirectly deteriorate pasture quality when flocks consume less roughage. Creep feeding and creep grazing in subtropical regions can also be important alternatives for improving lamb growth and GIN parasitic control.

GIN control in sheep, especially lambs, continues to be a challenge in subtropical regions. In these warm, high rainfall climates, resting pastures between grazing cycles to reduce GIN infection may take too long to be economically viable. However, *H. contortus* L3 larvae presence at various pasture canopy heights indicates that it is difficult to control their ingestion by lambs solely by manipulating grazing heights. Instead, deleterious effects of gastrointestinal parasitic reinfection from pasture can be reduced with improved lamb nutrition.

## Author's Note

The studies summarized in this manuscript were developed by Animal Science Graduate Programs of two universities in Brazil: Universidade Federal do Paraná and Universidade Federal do Rio Grande do Sul.

## Author Contributions

Every author had important contributions on this manuscript. The first CP and the second authors AM are the head of the research projects, being responsible for all parts of the studies. The third author is a researcher TD, and the fourth, fifth, and sixth authors FA, JPM, and LB are Ph.D. students who developed the research review. The seventh author JPM is a professor who contributed to manuscript configuration and publication. All authors contributed to the article and approved the submitted version.

## Conflict of Interest

The authors declare that the research was conducted in the absence of any commercial or financial relationships that could be construed as a potential conflict of interest.
